# Tumor Necrosis Factor Family Members and Myocardial Ischemia-Reperfusion Injury: State of the Art and Therapeutic Implications

**DOI:** 10.3390/ijms24054606

**Published:** 2023-02-27

**Authors:** Antonella Galeone, Maria Grano, Giacomina Brunetti

**Affiliations:** 1 Department of Surgery, Dentistry, Pediatrics and Gynecology, Division of Cardiac Surgery, University of Verona, 37129 Verona, Italy; 2 Department of Precision and Regenerative Medicine and Ionian Area, University of Bari Aldo Moro, 70124 Bari, Italy; 3 Department of Biosciences, Biotechnologies and Environment, University of Bari Aldo Moro, 70125 Bari, Italy

**Keywords:** tumor necrosis factor family, myocardial ischemia-reperfusion injury, myocardial infarction

## Abstract

Ischemic heart disease is the principal cause of death worldwide and clinically manifests as myocardial infarction (MI), stable angina, and ischemic cardiomyopathy. Myocardial infarction is defined as an irreversible injury due to severe and prolonged myocardial ischemia inducing myocardial cell death. Revascularization is helpful in reducing loss of contractile myocardium and improving clinical outcome. Reperfusion rescues myocardium from cell death but also induces an additional injury called ischemia-reperfusion injury. Multiple mechanisms are involved in ischemia-reperfusion injury, such as oxidative stress, intracellular calcium overload, apoptosis, necroptosis, pyroptosis, and inflammation. Various members of the tumor necrosis factor family play a key role in myocardial ischemia-reperfusion injury. In this article, the role of TNFα, CD95L/CD95, TRAIL, and the RANK/RANKL/OPG axis in the regulation of myocardial tissue damage is reviewed together with their potential use as a therapeutic target.

## 1. Introduction

**Figure 1:**
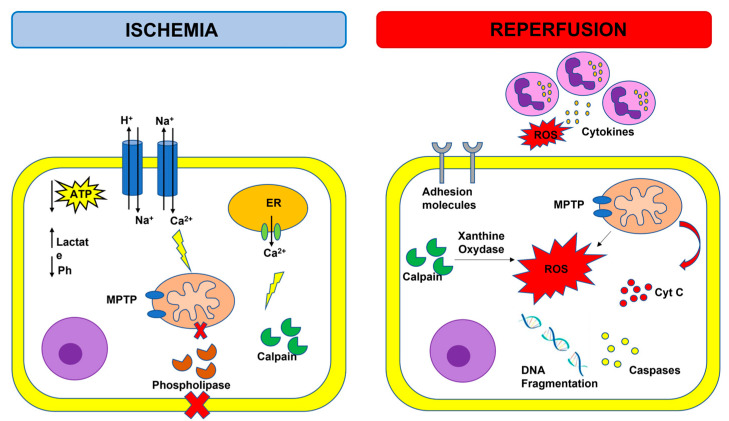
Overview of ischemia-reperfusion injury. Adenosine triphosphate (ATP); cytochrome C (cyt C); endoplasmic reticulum (ER); mitochondrial permeability transition pore (MPTP); reactive oxygen species (ROS); purple cells: neutrophils.

**Figure 2:**
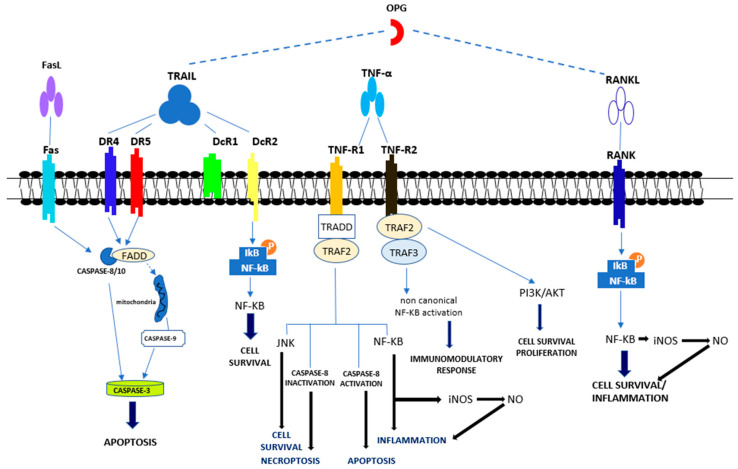
Overview of apoptotic and non-apoptotic signaling by the Tumor Necrosis Factor Receptor (TNFR)s. Apoptotic signal included the activation of caspase-8, -3 and -9. Pro-survival signal occurred through the activation of NF-κB, JNK, PI3K/AKT Abbreviations: Fas ligand (FasL); Tumor necrosis factor (TNF)-related apoptosis-inducing ligand (TRAIL); Death receptor 4 (DR4); Death receptor 5 (DR5); Decoy receptor 1 (DcR1); Decoy receptor 2 (DcR2); Tumor necrosis factor α (TNFα); Tumor necrosis factor receptor 1 (TNF-R1); Tumor necrosis factor receptor 2 (TNF-R2); Receptor activator of NF-κB (RANK) ligand (RANKL); Receptor activator of NF-κB (RANK); FAS-associated death domain (FADD); Tumor necrosis factor receptor type 1-associated death domain (TRADD); TNF receptor-associated factor 2 (TRAF2); TNF receptor-associated factor 3 (TRAF3); c-Jun N-terminal Kinase (JNK); Nuclear factor-κB (NF-κB); PhosphatidylInositol 3-kinase (PI3K); Protein kinase B (AKT).

Ischemic heart disease is the principal cause of death worldwide and clinically manifests as myocardial infarction (MI), stable angina, and ischemic cardiomyopathy [1]. Myocardial ischemia is usually due to coronary atherosclerosis and occurs when coronary blood flow is reduced because of the occlusion of a coronary artery or a deleterious redistribution of blood flow away from a given vascular territory [2]. Myocardial infarction is defined as an irreversible injury due to severe and prolonged myocardial ischemia inducing myocardial cell death. Type 1 MI is caused by atherothrombotic coronary artery disease and is consequent to the erosion or rupture of an epicardial coronary artery atherosclerotic plaque, followed by thrombosis and occlusion of the coronary artery. Myocardial injury caused by a mismatch between oxygen supply and demand and not by acute atherothrombotic plaque disruption is called type 2 MI [3]. Prompt and effective revascularization may reduce the loss of contractile myocardial muscle mass, decrease the infarct size, and improve clinical outcome [4]. In fact, infarct size is considered one of the major determinants of the prognosis of patients with acute MI [5]. Reperfusion rescues ischemic myocardium from cell death but also induces an additional irreversible injury known as myocardial ischemia-reperfusion (I/R) injury [6]. The pathological mechanisms of myocardial I/R injury that cause irreversible cell death include intracellular calcium overload, oxidative stress, endoplasmic reticulum stress, energy metabolism disorder, apoptosis, pyroptosis, ferroptosis, necroptosis, autophagy and inflammation [7] (Figure 1). The purpose of this review is to update the current knowledge regarding the involvement of tumor necrosis factor (TNF) and TNF super family (TNFSF) members in myocardial ischemia-reperfusion injury and the possible therapeutic implications (Figure 2).

## 2. Myocardial Ischemia-Reperfusion Injury

Activation of several innate immune molecular pathways have been observed in a spectrum of ischemic cardiac diseases including, but not limited to, infarction, I/R injury, post-injury left ventricular (LV) scaring, and LV dysfunction. Specifically, inflammatory response, mitochondrial damage and calcium overload, as well as cell death and cell survival-associated signaling pathways are involved in the pathophysiology of myocardial I/R injury [8]. During acute myocardial ischemia, ischemic cardiomyocytes switch to anaerobic metabolism to provide ATP, leading to lactate, H^+^, and nicotinamide adenine dinucleotide (NADH^+^) accumulation and cytosolic pH decrease. To reestablish the acid-based balance, the plasmalemma Na^+^/H^+^ exchanger is activated to extrude H^+^, and results in intracellular Na^+^ overload, which, in turn, activates the Na^+^/Ca^2+^ exchanger that leads to Na^+^ extrusion and intracellular Ca^2+^ overload [9]. The endoplasmic reticulum also reduces Ca^2+^ reuptake, which exacerbates intracellular Ca^2+^ overload. The elevation of intracellular calcium levels induces the opening of the mitochondrial permeability transition pore (MPTP) together with the activation of phospholipases and calpain, a Ca^2+^-dependent cysteine protease [10]. Reperfusion reestablishes blood supply in an ischemic area and provides an influx of oxygen that fuels the production of reactive oxygen species (ROS), which are harmful to the ischemic myocardium. Reperfusion after ischemia can result in injury rather than protection, and this phenomenon is called the oxygen paradox [11]. Calpain-induced xanthine oxidase formation, NADPH oxidase due to neutrophil respiratory burst, and damage to the mitochondrial electron transport chain may all contribute to the increase in ROS levels. The excessive production of ROS decreases membrane fluidity, increases calcium permeability, aggravates intracellular calcium overload and mitochondrial damage by opening the MTPM, and contributes to the release of pro-apoptotic factors, such as cytochrome C. ROS can react with proteins, cause loss of original protein structure and function, as well as damage nucleic acids and chromosomes. ROS also trigger the inflammatory system and cause the expression of cytokines and adhesion molecules that result in leukocyte aggregation, endothelial cell (EC) swelling, and contribute to the no-reflow phenomenon that indicates inadequate myocardial perfusion at the microvascular level even though the proximal coronary artery has been re-opened after a period of ischemia [12]. In response to myocardial ischemia, the inducible nitric oxide synthase (iNOS) is upregulated, leading to enhanced production of NO [13]. High levels of iNOS-derived NO are associated with an increased level of intracellular cGMP, resulting in a decrease in Ca^2+^ influx which depresses myofilament sensitivity to Ca^2+^ and, subsequently, attenuates cardiac contractile function [13]. NO also contribute to the formation of peroxynitrite, which subsequently leads to significantly increased oxidative stress and apoptosis, as well as the expression of pro-inflammatory cytokines [13].

Myocardial infarction is the result of cardiomyocyte necrosis, a type of cell death involving mitochondria and sarcolemma rupture, cell swelling, and the release of cellular debris activating inflammation. Cell damage and cell death lead to the release of cellular components such as heat shock proteins, high mobility group box-1, adenosine triphosphate, nuclear and mitochondrial DNA, and RNA into the extracellular space and the circulation. These molecules act as so-called damage (or danger)-associated molecular patterns (DAMPs) and serve as ligands for pattern recognition receptors (PRRs) that, when activated, induce nuclear translocation of various transcription factors as NF-κB and promote pro-inflammatory cytokine expression [14]. The involvement of more regulated forms of cardiomyocyte cell death has been recognized in I/R injury, including apoptosis, necroptosis, and pyroptosis [15,16].

Apoptosis occurs through the intrinsic pathway, following DNA damage, augmented ROS, and intracellular Ca^2+^ levels, or through the extrinsic pathway, following the activation of sarcolemmal death receptors. The process of apoptosis needs energy, includes the release of cytochrome C from mitochondria, and the activation of caspases, thus leading to DNA fragmentation. Apoptosis does not elicit an inflammatory reaction because the sarcolemma remains intact [17,18]. Opening of the MPTP, with consequential mitochondrial matrix swelling and outer membrane damage, has a major involvement in cardiomyocyte death [19,20]. Cytochrome C release following MPTP activation appears to be the main apoptosis-inducing mechanism [21]. The apoptosis level is also linked to the reperfusion duration. Prolonged periods of myocardial ischemia are linked to an increase in the necrosis rate, whereas, paradoxically, reperfusion leads to the increase in apoptosis. Reperfusion reestablishes glucose and oxygen supply, which is crucial for the survival of viable cells, but also reestablishes the energy required for apoptosis completion and might accelerate the apoptotic process [22,23]. Experimental studies in animals show that apoptosis can be triggered by ischemia and accelerated by reperfusion. Apoptosis is induced following 2 h of coronary occlusion and accelerated after 45 min of ischemia followed by 1 h of reperfusion [24,25]. Other studies in animals report apoptosis in myocardium exposed to a short-term period of ischemia followed by reperfusion, but not in the permanent ischemic area without reperfusion, suggesting that reperfusion initiates apoptosis [26,27].

Necroptosis follows the activation of sarcolemmal TNF receptors or toll-like receptors, which interact with specific serine/threonine-protein kinases and induces the formation of the necrosome. Necroptosis is characterized by the formation of pores in the sarcolemma and the premature loss of plasma membrane integrity, organelle swelling, and leakage of intracellular contents [28,29].

Pyroptosis starts with DAMPs, which lead to the formation of the inflammasome complex that triggers caspase activation, with the consequent formation of gasdermin-dependent pores in the sarcolemma [30,31].

Caspase-3 is known as a marker and key molecule of apoptosis; recent studies have also demonstrated its involvement in pyroptosis. TNFα stimulates caspase-3 to specifically cleave gasdermin E (GSDME), thus leading to the release of the N-terminal PFD of GSDME. The oligomerized N-terminal PFD of GSDME migrates towards the cell membrane to form non-selective pores, thus determining cell pyroptosis [32]. Necroptosis and pyroptosis finally induce the loss of plasma membrane integrity, thus eliciting a pro-inflammatory response with release of interleukins (ILs) and DAMPs. How and to what extent apoptosis, necroptosis, and pyroptosis interact/work in the context of myocardial I/R requires further investigation. Experimental studies in animals have shown that combined inhibition of necroptosis and apoptosis reduces infarct size more evidently than inhibition of either cell death type alone [32,33].

### 2.1. Tumor Necrosis Factor Alpha (TNFα)

TNFα, a member of the TNF superfamily, is a pro-inflammatory cytokine, initially identified as an inducer of cell death in tumor cells [34]. It is produced primarily by monocytes/macrophages, but B and T lymphocytes, natural killer cells, mast cells, neutrophils, fibroblasts, and osteoclasts can also secrete TNFα. It is initially synthesized as a 26 kDa homotrimer transmembrane protein (mTNF), where it either remains or is cleaved by a membrane-bound metalloproteinase known as TNF-converting enzyme (TACE) to produce the 17 kDa soluble TNF (sTNF) form. Following cleavage, sTNF is released into the blood plasma [35]. Membrane bound and soluble TNF can bind two receptors: TNFR1, which is expressed by all human tissues, and TNFR2, which, in contrast, is expressed primarily by immune cells, ECs, and neurons [36,37]. mTNF-TNFR2 binding generates a more effective response than sTNF [38]. TNFR1 and TNFR2 show different intracellular structures that bind several adaptor proteins [39]. The TNFR1 cytoplasmic tail includes the death domain (DD), thus leading it to engage the TNFR1-associated DD (TRADD) [40]; by comparison, TNFR2 recruits TNFR-associated factor (TRAF) 1 and 2 proteins [41]. The TNFR1 and 2 signaling pathways may trigger a cell survival response, whereas TNFR1 can also induce cell death based on the predominant physiological conditions, which are not completely known [42]. Other studies have led to crucial progress in the clarification of mechanisms regulating the crosstalk between TNFR1 and 2 together with the distinct, but complementary, roles of these two receptors [43,44].

TNFR1 activation can lead to the establishment of four signaling complexes, known as complexes I, IIa, IIb, and IIc, which are involved in different cellular reactions [44,45]. During complex I formation, the activated TNFR1 interacts with TRADD and other components resulting in the activation of mitogen-activated protein kinases (MAPKs) and NF-κB [46,47]. NF-κB dimers are normally present as an inactive form in the cytoplasm of cells because they are linked to members of the inhibitory family of IκB proteins. Following cell stimulation, IκB proteins are quickly phosphorylated, ubiquitinated, and then degraded, thus leading to the exposure of a nuclear localization sequence for the NF-κB proteins (Figure 1). NF-κB dimers thus migrate to the nucleus where they bind to specific sequences, termed κB sites, and, together with other transcription factors, regulate gene transcription. This finally determines the development of pro-survival signaling, where inflammation and immune cell proliferation are induced. Complex I signaling is fundamental for inflammation development, tissue degeneration, cell proliferation, and survival, as well as immune defense against pathogens [45,48]. In contrast to complex I, which is assembled in the cell membrane, complexes IIa, IIb, and IIc are assembled in the cytoplasm [49]. Complex IIa comprises TRADD, TRAF2, RIPK1, cIAP1/2, Fas-associated protein with death domain (FADD), and pro-Caspase-8, [50,51]. Complex IIb also includes RIPK3. The creation of complexes IIa and IIb, also recognized as apoptosome, trigger the activation of caspase-8, thus leading apoptosis. Complex IIc, which is also known as necrosome, triggers the mixed lineage kinase domain-like protein (MLKL) and causes/leads to inflammation and necroptosis [34,52].

TNFR2 engages TRAF2, together with TRAF1, cIAP1, and cIAP2, and this complex determines the downstream activation of NF-κB, AKT, and MAPKs, [49]. TNFR2 engagement is mainly linked to tissue regeneration, cell survival, and proliferation [53]. Furthermore, the activation of this pathway can trigger pro-inflammatory reactions. In general, TNFR1 is fundamental to determining pro-inflammatory and cytotoxic TNFα responses, whereas TNFR2 may be involved in cell proliferation, migration, or activation.

TNFα is involved in the pathogenesis of cardiovascular diseases, such as acute myocardial infarction [54], chronic heart failure (HF) [55], atherosclerosis [56], viral myocarditis [57], cardiac allograft rejection [58], and sepsis-induced cardiomyopathy [59].

The heart represents a TNF-producing organ, and both cardiac myocytes and myocardial macrophages produce it [60]. TNFα is not expressed in normal cardiac myocytes, but human cardiac myocytes expose a functional TNFR1 on their membrane and trigger an active response following TNFα binding [61]. Although originally described exclusively as a lipopolysaccharide (LPS)-induced macrophage cytokine, several studies indicate that cardiac myocytes synthetize an important quantity of TNFα following ischemia or LPS exposure. Certainly, ischemia-provoked myocardial TNFα production is significantly higher than sepsis-induced myocardial TNF production, and it may contribute to post-ischemic myocardial alteration by the inhibition of contractility as well as the triggering of myocyte hypertrophy and apoptosis [60]. The expression of TNFR1 and 2 also increases significantly after myocardial infarction [62], and it is positively correlated with infarction size, LV dysfunction, and remodeling [63].

LPS and ischemia-reperfusion activate myocardial p38MAPK and NF-κB with consequent TNFα production. This cytokine negatively affects myocardial function through mechanisms that are NO-dependent or sphingosine-dependent; furthermore, TNFα-TNFR1 interaction may induce cardiac myocyte apoptosis [64]. Experimental studies show that administration of exogenous TNFα reduces cardiac contractility in animals in a dose-dependent manner. TNFα reduces Ca^2+^ uptake by sarcoplasmic reticulum as well as myofilament Ca^2+^ sensitivity through the activation of p38MAPK. TNFα also induces cardiac caspase-8 activation, with consequent production of myocardial NO and mitochondrial ROS, thus resulting in ryanodine receptor S-nitrosylation and sarcoplasmic reticulum Ca^2+^ leak [65]. In vivo studies have demonstrated that TNFα also induces a hypertrophic response in cardiac myocytes by activation of NF-κB and p38MAPK through ROS [66]. In vitro studies have shown that cardiac myocytes undergo apoptosis after stimulation with TNFα, and that cardiac cell death is mediated by TFNR1. TNFR1, and not TNFR2, is mainly and highly expressed by cardiac myocytes in normal human hearts. TNFα stimulation also induces upregulation of TNFR2 that mediates cell repair [67].

Inflammation is recognized as the initial step of myocardial ischemia-reperfusion that leads to increased release of proinflammatory mediators, such as TNFα, IL1β, IL-2, IL-6, and IFN-α. TNFα has pleiotropic effects and can augment the local release of other pro-inflammatory mediators, including IL-1 and IL-6. TNFα shows both beneficial and harmful functions in the myocardium during I/R injury, depending on its concentration, receptor subtype, and exposure duration.

Ischemia and anoxia activate cardiomyocytes and myocardial local mononuclear macrophages to synthetize elevated amounts of TNFα, and, simultaneously, TNFR2 expression is also significantly augmented [62]. The TNFα–TNFR1 complex is primarily involved in the inflammatory response and ventricular remodeling after MI, and induces cardiomyocyte apoptosis and cardiotoxicity, whereas the TNFα-TNFR2 complex blunts these events after MI, reduces cardiomyocytes apoptosis, and exerts a protective effect on the heart [63]. After MI in myocardium, TNFα exerts a double function that is time- and dose-dependent. In particular, in the short term, low doses of TNFα could exert a protective role on the myocardium, whereas, in the long term, exposure to elevated TNFα secretion displays lethal activity on cardiomyocytes [68]. TNFα/TNFR1 interaction leads to FADD and TRADD secretion as well as inflammatory mediator release, which determines the progression of ventricular remodeling. TNFα/TNFR1 interaction determines the secretion of RIP1 which could be blocked by TAK1 activation [69]. TNFα /TNFR1 interaction can trigger the NF-κB pathway, stimulate ECs to expose VCAM-1 and ICAM-1, augment neutrophil infiltration into the infarction area, and also determine late ROS generation. TNFα/ TNFR2 interaction also activates NF-κB, but the expression of IL-6 and IL-1 β is inhibited to decrease the injury arising from the inflammatory status.

Ischemia/reperfusion injury or no-reflow frequently occurs during reperfusion after MI. This phenomenon is strictly linked with TNFα and clinically manifests with myocardial stunning, arrhythmia, microvascular injury, LV systolic dysfunction, and myocardial necrosis. The physio-pathological mechanisms comprise elevated Ca^2+^ accumulation in cardiomyocytes, high amounts of ROS production, and oxidoreductase activation. TNFα /TNFR1 interaction leads to NO synthesis, with consequent reduction of myofilament sensitivity to Ca^2+^ or activation of sphingomyelinase to reduce Ca^2+^ release. TNFα can also trigger the NF-κB pathway through TNFR1, thus resulting in a vicious cycle involving TNFα and other pro-inflammatory cytokines, which further exacerbate the injury. Experimental studies in animals have demonstrated the existence of sex differences in TNF signaling by TNFR1 after myocardial I/R. TNFR1 signaling resistance in females seems to allow a better postischemic recovery in female WT mice than in male WT mice. Additionally, TNF infusion induces less myocardial depression in female WT mice, despite equivalent TNFR1 expression. TNFR1 ablation positively affected postischemic myocardial function, reduced the activation of p38MAPK, and decreased IL-1β and -6 expression in males but not in females. Moreover, after I/R, WT females produced high levels of the suppressor of cytokine signaling protein 3, which can be partially linked to the TNFR1 signal resistance in the female myocardium [70]. Sex variances also occur in TNF/TNFR2 signaling. In particular, in isolated female and male murine hearts exposed to 20 min ischemia with subsequent 60 min reperfusion, TNFR2 deficiency led to reduced postischemic myocardial retrieval in both sexes, with a greater intensity in females. The negative effects of TNFR2 deficiency are linked to the reduced expression of SOCS3, STAT3, and vascular endothelial growth factor together with the enhanced expression of myocardial IL-1β synthesis in female hearts [71].

### 2.2. CD95L/CD95

CD95 ligand (CD95L also known as FasL, CD178, or TNFSF6), encoded by the *FASLG* gene, is a type II transmembrane protein displaying a transmembrane domain, a stalk region, a long cytoplasmic domain, a C-terminal region implicated in the CD95 binding, and a TNF homology domain (THD) involved in homotrimerization. The transmembrane CD95L may be cut in the stalk region by different matrix metalloproteases [72], thus resulting in the soluble form of CD95L (sCD95L), a homotrimer [73] whose interaction with CD95 fails to trigger cell death [74,75].

CD95, encoded by the *FAS* gene, is a tumor necrosis family receptor (TNF-R) member. In the cell membrane, CD95 auto-aggregates as a homotrimer, which is compulsory to increase cell death, and quickly assembles larger signaling platforms in the presence of CD95L [76]. CD95L/CD95 bonding leads to the engagement of FADD, which, consequently, binds pro-caspase-8 in the DISC complex [77]. Outside DISC assembly and activation of the apoptotic signal, FADD and caspase-8 are involved in the organization of different complexes involved in necroptosis or pyroptosis induction. In brief, RIPK1 ubiquitination is a key post-translational modification for the stimulation of NF-κB activation through TNF-R1 [78,79], and its deubiquitination determines cell death. The deubiquitinated RIPK1 recruits TRADD, pro-caspase-8, and FADD, together with the long isoform of FLICE-like inhibitory protein (FLIPL), to activate the apoptotic process [50]. In this complex, the caspase-8-mediated cleavage of RIPK1 obscures the kinase activity. Additionally, c-IAP1 and c-IAP2 degradation inhibits RIPK1 ubiquitination [80] and determines the assembly of another complex in which FADD, together with pro-caspase-8 and FLIPL, interact to activate the apoptotic process.

Once caspase-8 has been inactivated in these two complexes, it is possible to have the formation of the necrosome. In detail, RIPK1 recruits and activates RIPK3 to generate the necrosome; MLKL is a constitutive binding partner of RIPK3, and thus it is incorporated in the necrosome. MLKL phosphorylation leads to a conformational change, recruitment into the plasma membrane, and induction of necrosis through membrane permeabilization [81].

Ex vivo studies based on an I/R model of isolated rat and mouse hearts in Langendorff perfusion showed that caspase-dependent apoptosis occurs during postischemic reperfusion. Soluble CD95L is produced de novo and secreted by the postischemic hearts early after reperfusion onset. In primary adult rat myocyte culture, reoxygenation and hypoxia determined a strongly augmented sensitivity to CD95L apoptotic action. Isolated hearts from mice lacking functional CD95 (lpr) display a strong decrease in cellular death following ischemia and reperfusion with respect to wild-type mice [82]. Conversely, CD95 or CD95L deletion failed to decrease the myocardial infarct size in a Langendorff model of I/R injury, suggesting that the CD95 and CD95L apoptotic pathway is not the primary cause of myocardial infarct size and ventricular dysfunction caused by I/R injury [83]. In patients with MI, soluble CD95 was significantly augmented from baseline to 24 h, whereas CD95L reduced over time [63]. However soluble CD95 and CD95L did not show any correlation with infarct size, LV dysfunction, or measures of remodeling [63,84].

### 2.3. TRAIL

TRAIL, belonging to the TNF superfamily (TNFSF10), is a type II transmembrane protein, the active form of which is organized as a homotrimer. TRAIL expression has been demonstrated primarily in immune cells, but also in other tissues, including vascular, valvular, and ECs [85,86,87,88,89,90]. TRAIL determines its effect following binding with its multiple receptors. Five receptors are known for TRAIL (TRAIL-R): the death and the decoy receptors, respectively, DRs and DcRs. TRAIL-R1 (DR4) and TRAIL-R2 (DR5) with agonistic activity belonging to type I transmembrane proteins and show an intracellular death domain (DD) that promotes the apoptotic process (Figure 2). The DcRs with antagonist activity are represented by the soluble osteoprotegerin (OPG) as well as the transmembrane TRAIL-R3 (DcR1) and TRAIL-R4 (DcR2). DcR1 and DcR2 are proteins which do not have a fully developed intracellular DD. When TRAIL engages DR4 or DR5, it triggers a signaling pathway leading to apoptosis through extrinsic or intrinsic pathways. The assembly of the extrinsic pathway is characterized by the binding of DR4 and/or DR5 to the death-inducing signaling complex (DISC), which causes an increase in FADD, which is an intermediate complex involving DD and the inactive pro-caspase 8. Suddenly, the formation of active caspase 8 occurs, which leads to the activation of executive caspases (caspases 3, 6, and 7) with consequent cell apoptosis [91]. In some cells, the executive caspase activation must be additionally increased by the involvement of the internal mitochondrial apoptotic pathway, which is known as the intrinsic apoptotic pathway [88,92]. As for the DcRs for TRAIL, DcR1 is linked to the cell membrane through a glycosylphosphatidylinositol (GPI) linker and does not have a cytoplasmic domain, whereas DcR2 displays a shortened DD. The engagement of DcR2 can activate the NF-κB pathway that determines the transcription of genes promoting cell survival as well as apoptosis resistance (Figure 2) [93]. DcRs do not activate an apoptotic pathway when linked to TRAIL; they compete with DRs for TRAIL binding, thus exerting a protective mechanism against the pro-apoptotic effect of TRAIL [88]. The pro-apoptotic effect of TRAIL is primarily associated to neoplastic cells, or virus infected cells [87,88,92], but is also evident in normal cells [94,95,96]. It has also been shown, however, that TRAIL interaction with DR4 and DR5 can lead to the activation of survival pathways, such as ERK1/2 or PI3-kinase Akt [97]. Interestingly, transmembrane TRAIL stimulates DR4 and DR5 to the same extent, whereas soluble TRAIL mainly stimulates DR4 [98]. Consistently, DR5 is primarily expressed on normal cells, thus explaining their greater resistance to pro-apoptotic TRAIL effects. However, the triggering by TRAIL of the pathways activated by/activating or protected/protecting from apoptosis is linked to the cell type as well as to the balanced expression of death and decoy receptors. Cells resistant to TRAIL pro-apoptotic effects include VSMCs and ECs, although both cell types possess DR4 and DR5 [86,99].

It has been demonstrated in the literature that TRAIL is secreted from the postischemic heart shortly after reperfusion onset [82]. Experimental studies in animals indicate that DR5 is also up-regulated after MI, and that inhibition of TRAIL by blocking DR5 improves cardiac function after MI by preventing cardiac cell death and inflammation [100]. TRAIL can inhibit angiogenesis by determining ECs death but can also promote angiogenesis in vitro. Thus, TRAIL exhibits multiple and opposite effects that make its role in ischemic disease unclear. Experimental studies have shown that TRAIL stimulates angiogenesis following hindlimb ischemia in vivo. The TRAIL pro-angiogenic effect on human microvascular ECs is downstream from FGF2, with the involvement of NOX4 and NO signaling. These results have important therapeutic implications, such that TRAIL may ameliorate the angiogenic response to ischemia and augment perfusion recovery in patients with cardiovascular diseases [101].

### 2.4. RANKL/RANK/OPG Pathway

The receptor activator of NF-κB ligand (RANKL, TNFSF11) is a transmembrane protein, but a soluble form (soluble RANKL: sRANKL) is also detectable in the blood. This sRANKL derives from the cleavage of membrane-bound RANKL (mRANKL) by a metalloprotease. RANKL is encoded by the *TNFSF11* gene on chromosome 13. Trimers of mRANKL or sRANKL bind to RANK trimers following the interaction with specific proteins: TNFR-associated factor (TRAF) proteins. TRAFs are signaling transducers that bind the intracellular domains of various TNFRs. TRAF2 and TRAF6 are the most crucial for RANK signaling. RANK–RANKL signaling by TRAFs activates NF-κBs, mitogen-activated protein kinases (MAPKs), AP1, and interferon-regulatory factors (IRFs) [102]. RANKL is largely expressed on osteoblasts, osteocytes, infiltrating T cells and activated ECs. RANK is a type I transmembrane glycoprotein, and its gene is located on human chromosome 18q22.1. RANK is expressed on the cellular membrane of osteoclast precursors, osteoclasts, dendritic cells, B- and T-cells, chondrocytes, vascular endothelia, mammary gland epithelia, and bone marrow fibroblasts. RANKL exerts an important role in immune responses and osteoclastogenesis.

Osteoprotegerin (OPG, TNFRS11B) is a secreted glycoprotein of the TNF receptor superfamily encoded by the *TNFRSF11B* gene on chromosome 8 (8q24). Circulating measurable OPG exists either as a free 60 kD monomer or a disulfide bond-linked 120 kD homodimer form. The levels of OPG are gender-linked, with women showing greater OPG levels compared with men. Additionally, OPG levels are significantly linked with aging [103]. OPG is the soluble decoy receptor of RANKL and TRAIL. OPG interacts with RANKL through its N-terminal cysteine-rich domains (CRD), thus participating in bone homeostasis regulation. OPG binds TRAIL to regulate its pro-apoptotic activity. The crucial role of the TRAIL/OPG interaction is fundamental to inhibit TRAIL-induced apoptosis in different cell types [104].

OPG is expressed in various tissues, such as the heart, kidney, lung, liver, bone marrow, bone, and immune system, and is produced in vivo by osteocytes, osteoblasts, ECs, vascular smooth muscle cells (VSMCs), placenta, brain, and skeletal muscle [105,106]. OPG is synthetized in basal conditions by ECs following treatment with hormones, inflammatory cytokines, and various circulating molecules. IL-1β and TNFα have been demonstrated to augment OPG levels [107]. While RANKL and RANK are undetectable in healthy human vessels, OPG is expressed in normal arteries in coronary and aortic atherosclerotic plaques, and in the vicinity of VSMCs [103,108].

Various evidence suggests that besides its function in bone remodeling, signaling by the RANKL/RANK/OPG pathway is likewise involved in the pathophysiology of cardiovascular diseases, and it is actually considered one of the key regulators of the progression of calcification of the blood vessel wall [109,110,111,112,113,114,115,116,117,118,119]. Previous studies showed that serum sRANKL levels predict the cardiovascular event risk, including MI [120], and that RANKL may contribute to atherosclerotic plaque destabilization [121]. Additionally, it has been suggested that RANKL determines inflammation of the myocardium during acute cardiac overload [122] and induces impaired remodeling through matrix degradation after acute MI [123]. Studies in vitro showed that RANKL/RANK interaction determines the expression of IL-1α, IL-1β, and TNFα in cultured cardiomyocytes by activating the TRAF6-NF-κB pathway [120]. Experimental studies in mice subjected to 60 min of myocardial ischemia and different reperfusion times up to 72 h showed that RANKL amounts are increased during reperfusion both in systemic circulation and infarcted hearts, and intravenous post-infarction anti-RANKL treatments reduce infarct size and cardiac neutrophil infiltration [124]. In infarcted left ventricles, RANKL expression was significantly augmented by 12 to 72 h of reperfusion with respect to the baseline condition, while OPG protein expression did not change over time during reperfusion. Inside the infarcted hearts, OPG- and RANKL- positive regions were not co-localized, and OPG positivity was associated only to heart vessels. In mouse serum, RANKL levels had already significantly increased 5 min after reperfusion, with a peak observed at 12 h of reperfusion, while OPG serum levels were importantly decreased at 5 min and at 12 h after reperfusion [124]. Experimental studies showed that MI induced RANKL expression mainly in cardiomyocytes and scar-infiltrating cells in mice. In a highly manipulated murine model of myocardial ischemia (that did not include reperfusion), only selective inhibition of RANKL derived from hematopoietic cellular sources, but not selective inhibition of RANKL from mesenchymal cells, improved post-infarct survival and cardiac function. Curiously, a post-ischemic rise in LV gene expression of TNFα was not reduced by RANKL blockade in this study. The study concluded that RANKL produced by cells of hematopoietic origin, but not by cardiomyocytes, contributes to deteriorating cardiac function after MI [125]. Conversely, studies performed in patients with acute MI did not support the increase in RANKL serum levels demonstrated in mice, whereas an early increase in OPG serum levels was found [121,126]. Likewise, serum levels of OPG and T-cells, as well as monocyte gene expression of the NF-κB p50 subunit, significantly increase in patients undergoing coronary artery surgery [127]. Many studies have demonstrated a statistically significant increase in the levels of OPG and TNFα, together with the reduction of TRAIL amounts with the consequent increase in the OPG/TRAIL ratio in the plasma of patients in the acute phase of MI with respect to the controls [128]. An elevated plasma concentration of OPG and the OPG/TRAIL ratio are linked to significantly increased early (30-day) and late (1-year) mortality in patients with both ST and non-ST-segment elevation MI [129,130]. High levels of OPG and the OPG/TRAIL ratio are linked to adverse post-infarction LV remodeling and HF development after MI. In STEMI patients subjected to primary coronary angioplasty, a correlation has been found between the elevated plasma OPG levels on hospital admission and the no-reflow phenomenon frequency together with the appearing of adverse post-infarction LV remodeling [131]. Conversely, experimental studies suggest that OPG could exert a protective and pro-survival effect from oxidative stress in cardiomyocytes. Hydrogen peroxide (H_2_O_2_), an ROS, significantly increased the OPG production of adipose stem cells (ASC) and mRNA expression of OPG and DcR1, which attenuates TRAIL-induced apoptosis. In cardiomyocytes exposed to H_2_O_2_, treatment with ASC-derived OPG significantly improved cell viability by suppression of caspase 8 activation without affecting DR5 expression [132]. Thus, the function of the RANKL/RANK/OPG pathway in the setting of myocardial I/R injury has not been completely elucidated and requires further investigation.

## 3. Therapeutic Implications and Future Challenges

The research on TNFs leads to the identification of potential therapeutic targets (Table 1). The blockade of TNFα with etanercept 10 min prior to I/R injury improved cardiac functions, and reduced infarct size and cardiomyocyte apoptosis in mice [133]. Moreover, a single dose of etanercept injected at the time of MI improved long-term cardiac function and reduced cardiac tissue remodeling in rats [134]. The injection of anti-TNFα antibody 3 h prior to myocardial I/R has also been shown to reduce endothelial dysfunction by reducing the production of endothelial ROS [135]. In another study, a pharmacological TNFα inhibitor (CAS1049741-03-8), inhibiting binding the protein to its receptor, decreased post-infarction inflammatory response but negatively affected cardiac activity due to increased cardiomyocyte apoptosis [136]. Transgenic mice lacking one or the other TNFR leads to the demonstration that the majority of the cardioprotective activity involved TNFR2, while TNFR1 activation triggers pathogenic processes. Consistently, TNFR2 activation blocks the pathogenic TNFR1 downstream pathways. It has been reported that, in the absence of TNFR2, there is evident augmented activity of TNFR1 downstream effector molecules NF-κB [137] and p38MAPK [138] together with an augmented secretion of IL-1β and IL-6 [139]. This could explain the conflicting results obtained between human and animal studies. In fact, a single high dose injection of etanercept did not ameliorate patient outcomes following acute MI [140].

The documented key role of TNFα in cardiovascular events encouraged the testing of its therapeutic value in patients with systolic HF. Randomized, double-blind, placebo-controlled trials were aborted after failing to demonstrate a beneficial effect of etanercept in HF patients with reduced ejection fraction. In fact, the RECOVER (Research into Etanercept: Cytokine Antagonism in Ventricular Dysfunction) and RENAISSANCE (Randomized Etanercept North American Strategy to Study Antagonism of Cytokines) clinical trials were stopped in advance due to lack of beneficial effect [141]. Consistently, the Randomized Etanercept Worldwide Evaluation (RENEWAL) trial, combining the results of RECOVER and RENAISSANCE testing the efficacy and safety of etanercept, demonstrated the absence of helpful effects in terms of mortality and hospitalization [142]. Additionally, in the ATTACH (Anti-Tnf alpha Therapy Against Chronic Heart failure) short-term trial, TNFα antagonism using infliximab did not ameliorate, and high doses increased the risk of HF-related hospitalization or death of patients affected by moderate-to-severe chronic HF [143]. In addition, another study reported that a single high dose etanercept injection did not improve patients’ outcomes following acute MI [140]. Thus, in patients with systolic HF, continuous anti-TNFα treatment did not determine positive effects and can be detrimental and aggravate the disease. Consequently, the use of TNFα inhibitor is not recommended. Thus, in the failing heart, TNFα exerts a cardioprotective effect, but the mechanism should be further investigated. Differently, in patients with autoimmune inflammatory diseases, a long-term anti-TNFα therapy is usually not detrimental, and it can even protect from the risk of increased cardiovascular complications and death. TNFα antagonist use has been linked with a reduced risk of MI and development of acute coronary syndrome, highlighting anti-TNFα therapy as a promising anti-atherosclerotic therapy in rheumatoid arthritis patients (Table 1) [144,145]. It is important to remember that anti-TNFα therapy represents the leading treatment for rheumatic diseases. These patients frequently display a rapid development of diastolic function change. Patients with rheumatoid arthritis and preserved LV activity treated with infliximab displayed a cardiac function improvement [146] together with reduced LV torsion [147]. A large cohort of clinical studies has demonstrated the reduced cardiovascular-related death of rheumatoid arthritis patients treated with adalimumab, infliximab, or etanercept. In an additional multi-center comparative study in patients undergoing long term treatment with adalimumab, etanercept, and infliximab, a decreased risk of cardiovascular-related death was found with respect to patients receiving disease modifying antirheumatic drugs (DMARD). Similar findings have been reported for patients with psoriasis that are at high risk of developing cardiovascular diseases [148,149].

RANKL also contributes to post-MI injury and repair, and thus the anti-RANKL effect was tested in animal models of myocardial ischemia. During ischemia, a “one-shot” injection of neutralizing anti-RANKL IgG reduced MI size and improved cardiac function but did not affect adverse remodeling. These positive effects were associated in vivo with a decrease in cardiac neutrophil infiltration as well as MMP-9 and ROS release. Anti-RANKL IgG injection decreased the rapid increase in neutrophil granule enzymes and cytokines in serum after reperfusion onset [124].

## 4. Conclusions

Different studies have reported the involvement of TNFα, RANKL/RANK/OPG axis and TRAIL in MI, thus also stimulating studies on the effect of their neutralization. To date, the neutralization of TNFα in MI patients has not shown a reduction in cardiovascular events, nor an improvement in myocardial function. However, in patients with rheumatic disease, treatment with TNFα inhibitors shows a protective effect against cardiovascular diseases in comparison with other standard treatments. Few studies have been performed on RANKL inhibition, due to the discouraging results obtained in animal models, possibly because RANKL represents an intermediate of the cascade and not the initiator, or maybe because of the pro-survival signaling associated with RANKL. TRAIL seems to be involved in MI, but its signaling pathway is very complex due to the multiple receptors able to bind it; however, trials demonstrating the safety of molecules affecting TRAIL signaling are ongoing for the treatment of cancer and, in the future, could also be used for MI management. Indeed, additional molecular targets belonging to the TNF superfamily, such as tumor necrosis factor-like weak inducer of apoptosis (TWEAK) and CD40L, could give encouraging results. It is also important to remember that other cytokines, such as ILs, are involved in heart disease and myocardial I/R injury, and that the preliminary results of ongoing trials seem to be encouraging.

## Figures and Tables

**Table 1:** Summary of major experimental studies testing anti-TNFs.

Target	Intervention	Phenotype/Outcomes	References
TNFα	Single etanercept injection directly after MI (animal model of permanent coronary occlusion)	Reduced inflammation with improved remodeling	[135]
TNFα	Single infusion of etanercept in MI patients	Reduced systemic inflammation, augmented platelet activation	[140]
TNFα	Etanercept injection in patients with chronic heart failure (RENAISSANCE TRIAL)	Prematurely stopped due to the lack of positive outcomes	[141]
TNFα	Etanercept injection in patients with chronic heart failure: results of the Randomized Etanercept Worldwide Evaluation (RENEWAL)	The results of RENEWAL exclude a clinically relevant effect of etanercept on the rate of death or hospitalization	[141]
TNFα	The Randomized Etanercept Worldwide Evaluation (RENEWAL) trial combining the results of RECOVER and RENAISSANCE testing the efficacy and safety of etanercept in patients with chronic heart failure	No evidence of beneficial effects in terms of hospitalization or mortality	[142]
TNFα	Injection of infliximab in patients with heart failure multicenter, double bind trial Anti-Tnf alpha Therapy Against Congestive Heart failure (ATTACH)	Left ventricular ejection fraction amelioration after 14 weeks treatment with infliximab.	[143]
TNFα	Multi-center comparative study in rheumatoid arthritis patients treated with adalimumab, infliximab, or etanercept	Significantly reduced risk of myocardial infarction in comparison with patients receiving synthetic DMARD ^1^	[144]
TNFα	Multi-center comparative study in patients: long term treatment with adalimumab, etanercept, and infliximab	Decreased risk of cardiovascular-related death with respect to patients receiving DMARD ^1^	[145]
TNFα	Single center clinical trials involving 23 patients with rheumatoid arthritis receiving infliximab infusion every 2 months	Improvement of left ventricular fraction and decreased levels of pro-inflammatory cytokines	[146]
TNFα	Single center clinical trial involving 68 patients with rheumatoid arthritis treated for 180 days with infliximab or prednisolone	Amelioration of left ventricular radial and longitudinal systolic deformation and reduced left ventricular torsion were registered compared to patients treated with prednisolone	[147]
TNFα	Multicenter comparative study involving 8845 psoriatic patients, treated for at least two months with etanercept, adalimumab, or infliximab	Decreased risk of MI with respect to TNFα inhibitor naïve patients	[148]
TNFα	Multicenter comparative study involving 17,729 psoriatic patients, treated for 150 days with etanercept, adalimumab, infliximab, or methotrexate	Decreased risk of cardiovascular events in patients treated with anti-TNFα inhibitors with respect to methotrexate	[149]
RANKL	Animal model of myocardial infarction	Reduction of myocardial infarct size, without affecting remodeling ^1^	[124]

^1^ DMARD: disease modifying antirheumatic drugs.

## Data Availability

No new data were created or analyzed in this study. Data sharing is not applicable to this article.
